# The Cytotoxicity of Doxorubicin Can Be Accelerated by a Combination of Hyperthermia and 5-Aminolevulinic Acid

**DOI:** 10.3390/antiox10101531

**Published:** 2021-09-27

**Authors:** Hiromi Kurokawa, Hirofumi Matsui

**Affiliations:** 1 Algae Biomass Research and Development, University of Tsukuba, 1-1-1 Tennodai, Tsukuba 305-8577, Japan; hmatsui@md.tsukuba.ac.jp; 2 Faculty of Medicine, University of Tsukuba, 1-1-1 Tennodai, Tsukuba 305-8577, Japan

**Keywords:** doxorubicin, 5-aminolevulinic acid, hyperthermia, mitochondrial reactive oxygen species

## Abstract

Chemotherapy is cytotoxic to various cancer cells and as well as normal cells. Thus, treatments that demonstrate selective cytotoxicity for cancer cells are desired. The combination of chemotherapy and other cancer therapies can show synergic cytotoxicity, which may be a clue to the nature of the involved cancer cellar-specific damage. We previously reported a phenomenon whereby mitochondrial reactive oxygen species (mitROS) regulate the expression transporters involved in anticancer drug transport and mitROS production is increased by hyperthermia. Moreover, the uptake of 5-aminolevulinic acid (ALA) was enhanced by the increase in mitROS production. In this study, we investigated whether the combination of hyperthermia and ALA can enhance the cytotoxicity of doxorubicin. MitROS production and ALA-derived porphyrin accumulation by hyperthermia (HT) were increased in a murine breast cancer cell line. The expression of solute carrier 15A1 (SLC15A1) upregulated and an ATP-binding cassette subfamily G member 2 (ABCG2) downregulated by HT. Since SLC15A1 is an accumulating transporter for ALA, while ABCG2 is a porphyrin efflux transporter, porphyrin accumulation was enhanced. ABCG2 is also a doxorubicin efflux transporter. Thus, ALA treatment accelerates the intracellular concentration of porphyrin, which acts as a competitive inhibitor of doxorubicin. Indeed, the amount of intracellular doxorubicin was increased by a combination of HT and ALA. The cytotoxicity of doxorubicin was also enhanced. This enhancement was observed in the human breast cancer cell line while it was not seen in normal cells. The combination of HT and ALA treatment can enhance the cancer-specific cytotoxicity of doxorubicin.

## 1. Introduction

Hyperthermia (HT) is a cancer treatment strategy based on the different heat sensitivities of normal and cancer tissues [1]. This difference in sensitivities becomes high at a low pH, and under nutritional deprivation, cancer tissues are more sensitive to heat than normal tissues [2]. Vascular distribution also affects heat sensitivity. Normal tissues have enough vascular distribution to mitigate fever and attenuate tissue damage. In contrast, heterogeneity in the distribution of vasculature and blood flow increases the heat stress in cancer tissues [3]. Furthermore, recent studies have reported that HT includes six components: blocking cell survival, inducing cellular stress response, modulating immune response, evading DNA repair, changing tumor microenvironment, and sensitization to radiation and chemotherapy [4]. Because the effect of HT alone is not sufficient for cancer treatment, it is used in combination with conventional therapies [5]. The combination therapies not only exhibit synergistic effects, but also have fewer side effects because of the reduced drug dosages [6]. In open-label, nonrandomized clinical trials, combination therapy may be able to efficiently treat refractory or recurrent malignancies [7]. The effects of gemcitabine and cisplatin, as well as of gemcitabine and 5-fluorouracil combination chemotherapies, were reported to be enhanced when used together with HT treatment [8,9]. Moreover, the cytotoxicity of bleomycin, paclitaxel, and oxaliplatin was reported to be increased with HT treatment [10].

The most important problem with chemotherapy is the drug resistance of cancer cells [11]. It is known that resistant cells express transporters associated with altered drug permeability [12]. Many anticancer drugs are excreted from the cells by the action of ATP-binding cassette (ABC) transporters [13]. Some drug-resistant cancer cells, including cancer stem cells, overexpress ABC transporters and, therefore, they escape the cytotoxicity of anticancer drugs owing to insufficient intracellular drug concentrations [14,15]. Moreover, many patients with metastatic cancer die owing to drug-resistant cancer cells. Therefore, it is very important to regulate the expression of ABC transporters [12]. In addition to investigation of the regulation of ABC transporters, there are several reports concerning combination treatment using a competitive inhibitor. It is reported that competitive inhibitors can enhance the effect of anticancer drugs because the efflux transporter of the competitive inhibitor and the anticancer drug are the same [16]. Therefore, the competitive inhibitor can increase the concentration in the serum to enhance the cytotoxicity of anticancer drugs [17,18,19].

In this study, we investigated whether the effects of anticancer drugs could be enhanced by regulating the expression of ABC transporters using the competitive inhibitor. The combination treatment can accelerate the effect of anticancer drugs compared with treatments administered individually. To regulate the expression of ABC transporter, we selected pretreatment HT. We previously reported that ABCG2 expression is decreased transiently by pretreatment HT via increased production of mitochondrial reactive oxygen species (mitROS) [20]. Moreover, we selected 5-aminolevulinic acid (ALA) as a competitive inhibitor. ALA is a nonproteinogenic amino acid and a precursor of porphyrins [21]. Porphyrins are photosensitizers that accumulate specifically in cancer cells and are utilized as drugs in photodynamic therapy (PDT) [22]. Porphyrins and doxorubicin (DOX), an anticancer drug, are excreted by ABCG2 out of the cells. Thus, we hypothesized that the cytotoxic effect of DOX could be enhanced when it is used in combination with HT for the regulation of ABCG2 transporter with ALA as a competitive inhibitor.

## 2. Materials and Methods

### 2.1. Cell Culture and HT Treatment

The murine breast cancer cell line 4T1E was established by Dr. Okada [23,24]. The 4T1E cells were cultured in a high-glucose RPMI-1640 medium (Wako Pure Chemical Industries Ltd., Osaka, Japan). The human breast cancer cell line MDA-MB-231 was purchased from the American Type Culture Collection (Rockville, MD, USA). The MDA-MB-231 cells were cultured in DMEM. The rat gastric epithelial cell line RGM1 was purchased from RIKEN cell bank (Ibaraki, Japan). The RGM1 cells were cultured in DMEM/F12 (Life Technologies Japan Ltd., Tokyo, Japan). The culture media contained 10% inactivated fetal bovine serum (Biowest LLC, Kansas City, MO, USA) and 1% penicillin-streptomycin (Wako Pure Chemical Industries Ltd.). Cells were cultured in 5% CO_2_ and 37 °C. The cells were seeded in dishes or plates overnight before treatment. For HT treatment, the cells were incubated at 37 °C or 42 °C for 1 h. For recovery after the treatment, the cells were cultured in the cell culture incubator at 37 °C under an atmosphere of 5% CO_2_ until further analysis.

### 2.2. Electron Spin Resonance Spectroscopy

ROS generation in cells was measured by electron spin resonance (ESR). The 4T1E cells were seeded on a glass cover slide (49 mm × 5 mm × 0.2 mm) and incubated until they were confluent. The cells were incubated at 37 °C or 42 °C for 1 h. The cover slide, with cells attached, was placed in a glass holder and immersed in a respiratory buffer containing 5 mM succinate, 5 mM malate, 5 mM glutamate, 5 mM nicotinamide adenine dinucleotide, and 10 mM 5,5-dimethyl-1-pyrroline-N-oxide (DMPO; Dojindo, Tokyo, Japan). All ESR spectra were obtained using a JEOL-TE X Band spectrometer (JEOL Ltd., Tokyo, Japan) under the following conditions: 7.5 mT sweep width, 1000 gain, 0.1 mT modulation width, 0.1 s time contrast, 335.5 mT center field, 9.4 GHz frequency, and 10 mW incident microwave power.

### 2.3. Measurement of Mitochondrial ROS

MitROS was detected using a fluorescence indicator, MitoSOX (Life Technologies Japan Ltd.). The 4T1E cells were incubated overnight in 6-well plates at a density of 2 × 105 cells/well. The cells were incubated at 37 °C or 42 °C for 1 h and then incubated for 10 min in 5 μM MitoSOX diluted with MSF buffer containing 5.4 mM KCl, 136.9 mM NaCl, 8.3 mM glucose, 0.44 mM KH_2_PO_4_, 0.33 Na_2_HPO_4_, 10.1 mM HEPES, 1 mM MgCl_6_ 6H_2_O, and 1 mM CaCl_2_ 2H_2_O. After incubation, the cells were rinsed with MSF buffer and replaced with FluoroBrite DMEM. The fluorescence intensity of MitoSOX was measured using a Synergy H1 microplate reader (BioTek Instruments Inc., Winooski, VT, USA). The excitation and emission wavelengths used were 510 and 580 nm, respectively. Moreover, the intracellular fluorescence intensity of MitoSOX was measured with a fluorescence microscope IX83 (Olympus Optical Co Ltd., Tokyo, Japan). The fluorescence was excited at 535–555 nm and the emission was collected using a 570–625 nm filter.

### 2.4. Porphyrin Accumulation in Cells Treated with ALA

The 4T1E cells were incubated overnight in 12-well plates at a density of 1 × 105 cells/well. The cells were incubated at 37 °C or 42 °C for 1 h and then at 37 °C for 24 h. After the treatments, the cells were incubated with 1 mM ALA for 6 h and then rinsed with PBS and lysed in 100 μL RIPA buffer [25]. The cell homogenates were transferred to a 96-well plate. The fluorescence intensity of porphyrin, which was made from ALA, was measured using a Varioskan microplate reader (Thermo Fisher Scientific Inc., Waltham, MA, USA). The excitation and emission wavelengths were 409 and 634 nm, respectively. The fluorescence of intracellular ALA-derived porphyrin was determined using a fluorescence microscope. The cells were incubated overnight in a 35 mm glass bottom dish at a density of 1 × 105 cells. They were further incubated at 37 °C or 42 °C for 1 h and then at 37 °C for 24 h. The cells were subsequently treated with 1 mM ALA for 6 h, after which the supernatant was aspirated and replaced with MSF buffer. Fluorescence was detected at an excitation wavelength of 635–675 nm and an emission wavelength of 696–736 nm.

### 2.5. Western Blotting Analyses

The 4T1E cells were incubated overnight in 60 mm dishes. The cells were further incubated at 37 °C or 42 °C for 1 h and then at 37 °C for 24 h. They were then rinsed three times with PBS, and whole cell lysates were prepared by adding RIPA buffer containing a protease inhibitor cocktail (Thermo Fisher Scientific Inc., Waltham, MA, USA) on ice, and then boiling at 95 °C for 10 min. For SDS polyacrylamide gel electrophoresis, the cell lysates were loaded into the wells of NuPAGE^®^ Novex^®^ 4–12% Bis-Tris gels (Thermo Fisher Scientific Inc.). The gels were electrophoresed at 100 V for 60 min and proteins were transferred onto a PVDF membrane by electrophoresis at 2.0 mA/cm^2^ for 60 min. The membrane was blocked for 60 min with PVDF blocking reagent for the Can Get Signal^®^ (Toyobo Co. Ltd., Osaka, Japan) and probed with primary and secondary antibodies. Antirabbit SLC22A16 (cat. no. LS-C176922) (LSBio, Seattle, WA, USA), ABCG2 (cat. no. 42078) (Cell Signaling Technology Japan K.K., Tokyo, Japan), or SLC15A1 (cat. no. bs-10588R) (Bioss, Boston, MA, USA) antibodies (1:1000) were added to the Can Get Signal^®^ Immunoreaction Enhancer Solution 1 (Toyobo Co. Ltd., Osaka, Japan). The membrane was exposed to this solution overnight. After removal of the primary antibody solution, the membrane was washed three times with 1X Tris-buffered saline containing Tween 20. Thereafter, the secondary HRP-linked antirabbit IgG (Cell Signaling Technology Japan, K.K.; 1:1000) was added to the Can Get Signal^®^ Immunoreaction Enhancer Solution 2 (Toyobo Co. Ltd., Osaka, Japan) and the membrane was exposed to this solution for 60 min. Lumina Forte Western HRP substrate (Millipore Co., Billerica, MA, USA) was used to visualize the membrane. Images of the blots were captured using ImageQuant LAS 4000 (GE Healthcare Japan, Tokyo, Japan). β-Actin (Cell Signaling Technology Japan, K.K.) was detected as a control for protein loading.

### 2.6. Cellular Uptake of DOX

The 4T1E cells were incubated overnight in 6-well plates at a density of 2 × 10^5^ cells/well. They were further incubated at 37 °C or 42 °C for 1 h and then at 37 °C for 24 h. Thereafter, the cells were incubated with 0.1 μM DOX (Wako Pure Chemical Industries Ltd., Richmond, VA, USA) in the presence or absence of 1 mM ALA for 24 h. The cells were then rinsed with PBS and lysed in 100 μL RIPA buffer. The cell homogenates were transferred to a 96-well plate. The fluorescence intensity of DOX was measured using a Synergy H1 microplate reader. The excitation and emission wavelengths were 485 and 595 nm, respectively. The fluorescence of intracellular DOX was determined using a fluorescence microscope. The cells were incubated overnight in a 60 mm dish at a density of 2 × 10^5^ cells. They were further incubated at 37 °C or 42 °C for 1 h and then at 37 °C for 24 h. The cells were subsequently treated containing 0.1 µM DOX with or without 1 mM ALA for 24 h, after which the supernatant was aspirated and replaced with MSF buffer. Fluorescence was detected at an excitation wavelength of 460–495 nm and an emission wavelength of 575 nm.

### 2.7. Cell Viability Assay

Cell viability assay was performed using the Cell Counting Kit-8 (Dojindo, Rockville, MD, USA), according to the manufacturer’s protocol. The 4T1E and RGM1 cells were cultured on 96-well plates at a density of 2 × 10^3^ cells/well, and MDA-MB-231 was cultured on 96-well plates at a density of 5 × 10^3^ cells/well incubated overnight. The cells were subsequently incubated at 37 °C or 42 °C for 1 h and then at 37 °C for 24 h. After the treatment, the culture medium was aspirated out and replaced with a medium containing DOX, with or without 1 mM ALA. The cells were incubated at 37 °C for 24 h. These cells were then incubated with a 10% Cell Counting Kit-8. The absorbance was measured at 450 nm using the DTX 880 multimode microplate reader (Beckman Coulter Inc., Brea, CA, USA) or a Synergy H1 microplate reader.

### 2.8. Statistical Analysis

Data are expressed as means ± SD and were assessed by analysis of variance. Individual groups were compared with Tukey’s post hoc test or Student’s *t*-test with a value of *p* < 0.05 considered statistically significant.

## 3. Results

### 3.1. HT Induced Mitochondrial ROS Generation in Cancer Cells

**Figure 1:**
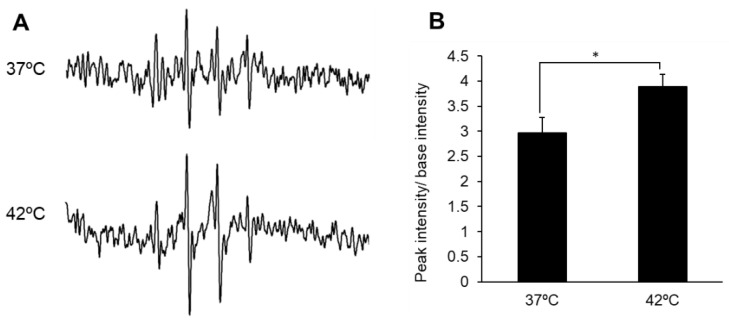
Intracellular ROS production after HT treatment. (**A**) The ESR signal of 4T1 cell; (**B**) The relative ESR signal. Data are expressed as means ± SD (*n* = 3). * *p* < 0.05. Student’s *t*-test.

**Figure 2:**
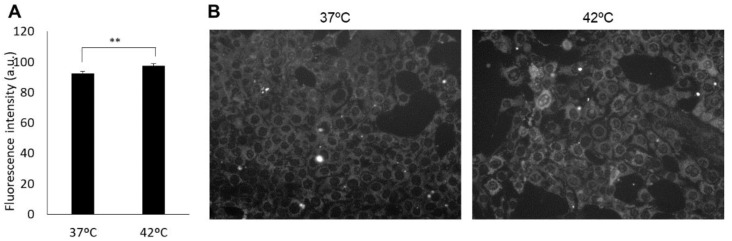
The fluorescence intensity of MitoSOX. (**A**) Intracellular fluorescence intensity detected using a microplate reader. (**B**) Fluorescent microscopy utilized to assess cellular uptake of MitoSOX. Data are expressed as means ± SD (*n* = 4). ** *p* < 0.01. Student’s *t*-test.

The spin adduct results from the trapping of superoxide and hydroxyl radicals with DMPO [26]. The ESR spectral intensity in 4T1E cells increased significantly after HT treatment (Figure 1A). In comparison with base intensity, the peak intensity increased significantly from 2.97 ± 0.30 to 3.89 ± 0.24 in HT treatment compared with that in the control (Figure 1B). MitoSOX is one of the fluorescent dyes that can detect mitROS. The results of the MitoSOX assay showed that HT increased the fluorescence intensity of MitoSOX and intracellular mitROS production was increased by HT (Figure 2A,B).

### 3.2. HT Enhanced Porphyrin Synthesis in Cells Treated with ALA

**Figure 3:**
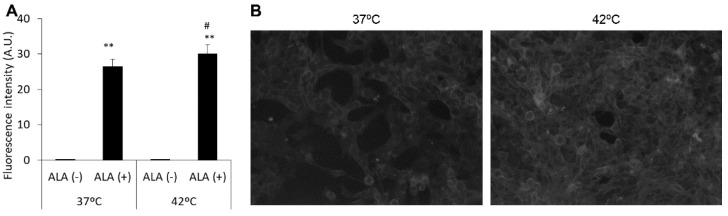
The fluorescence intensity of porphyrin-derived ALA. (**A**) Fluorescence intensity of porphyrin measured by a microplate reader; (**B**) Fluorescence intensity distribution in cells observed under the fluorescence microscopy. The cells were incubated at 37 °C or 42 °C for 1 h and then at 37 °C for 24 h. After the treatments, the cells were incubated with 1 mM ALA for 6 h. Data are expressed as means ± SD (*n* = 6). ** *p* < 0.01 vs. 37 °C ALA (−). # *p* < 0.05 vs. 37 °C ALA (+). Tukey’s post hoc test.

The fluorescence of porphyrin was barely detected in cells without ALA treatment. The porphyrin synthesis of these cells was not increased by HT treatment as compared with that in non-HT-treated cells. With respect to ALA-treated cells, porphyrin synthesis increased significantly in HT-treated cells (26.7 ± 0.94) as compared with that in cells that were not subjected to HT treatment (29.9 ± 1.3; Figure 3A). Actually, intracellular fluorescence intensity in HT treatment became higher than in the control (Figure 3B).

### 3.3. Expression of ABCG2 and SLC15A1, but Not of SLC22A16, Is Regulated by HT

**Figure 4:**
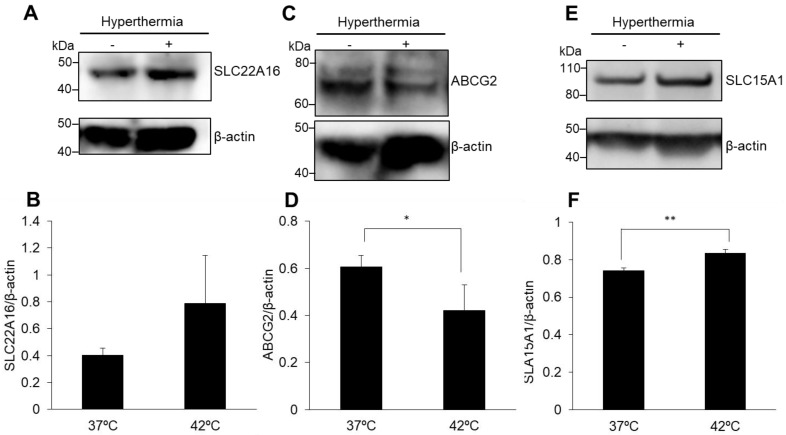
Western blot analysis of each transporter expression. SLC22A16 (**A**,**B**), ABCG2 (**C**,**D**) and SLC15A1 (**E**,**F**) were normalized to β-actin expression. Data are expressed as the mean ± SD (*n* = 3). * *p* < 0.05, ** *p* < 0.01. Student’s *t*-test.

The alteration of each transporter expression was analyzed by Western blotting. SLC22A16 is a DOX influx transporter. SLC22A16 expression was upregulated by HT; however, it was not significantly different from that in the non-HT treatment (Figure 4A,B). The expression of ABCG2 was significantly decreased by HT treatment compared with its expression in non-treated cells (Figure 4C,D). Similar to previous reports, SLC15A1 expression was significantly upregulated by increasing mitROS production via HT treatment (Figure 4E,F).

### 3.4. Intracellular DOX Accumulation Increased by HT and ALA Treatment

**Figure 5:**
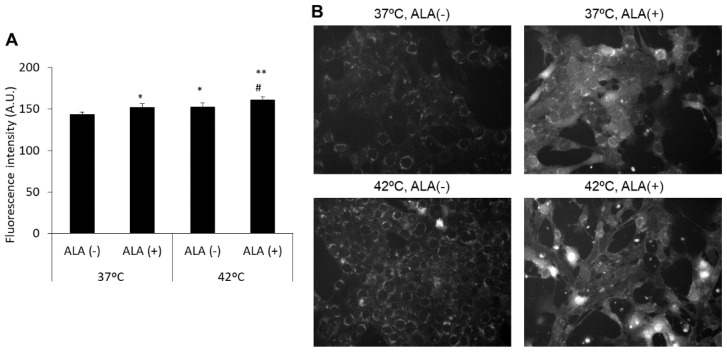
Fluorescence intensity of DOX. (**A**) Fluorescence intensity was measured using microplate reader. (**B**) Intracellular fluorescence intensity of DOX was observed under fluorescence microscopy. Data are expressed as means ± SD (*n* = 6). * *p* < 0.05 and ** *p* < 0.01 vs. 37 °C ALA (−). # *p* < 0.05 vs. 37 °C ALA (+). Tukey’s post hoc test.

Intracellular DOX fluorescence was found to be increased by ALA or HT treatment as compared with the control. There was no significant difference between ALA and HT. Moreover, the intracellular fluorescence intensity of the ALA and HT combination treatment was highest (Figure 5A). Fluorescence intensity in the ALA of HT was higher than that in the control. Fluorescence intensity showed that certain cells were higher in ALA, while all cells were uniformly higher in HT. Compared with the four groups, fluorescence intensity in ALA+HT was highest (Figure 5B).

### 3.5. Combination of HT and ALA Treatment Synergistically Enhanced the Cytotoxicity of DOX

**Figure 6:**
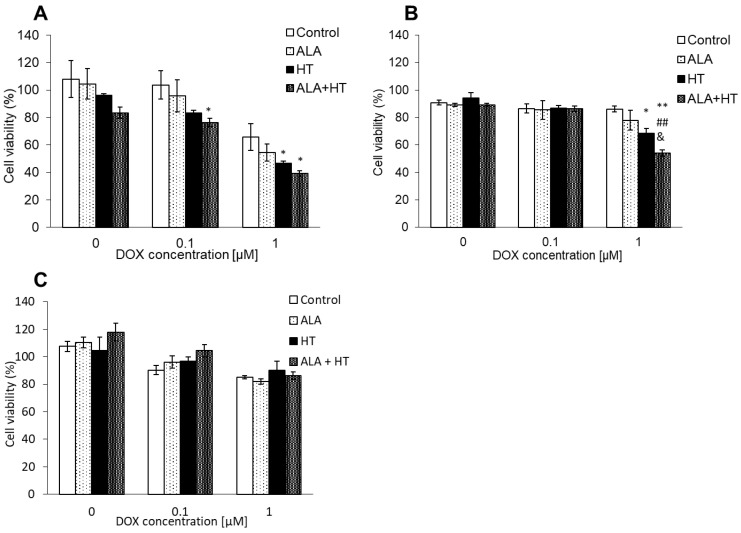
Cytotoxicity effect of DOX. (**A**) 4T1E; (**B**) MBA-MB-231; and (**C**) RGM1 cells. It was enhanced by ALA or HT treatment. ALA+HT treatment most enhanced the cytotoxicity of DOX. Data are expressed as the mean ± SD (*n* = 4). * *p* < 0.05 and ** *p* < 0.01 vs. Control. ## *p* < 0.01 vs. ALA. & *p* < 0.05 vs. HT. Tukey’s post hoc test.

The effect of a combination therapy of HT and ALA was determined. In 0.1 μM DOX treatment, the viability of cells treated with ALA and HT slightly decreased compared with the viability of control cells. The cytotoxicity of 1 μM DOX treatment was significantly enhanced in each group. In particular, the viability of cells in the ALA and HT treatment decreased to 39.3 ± 1.4% compared with that in the control (65.8 ± 9.8%) (Figure 6A). In addition, in human breast cancer MDA-MB-231, the cytotoxicity of 1 microM DOX was enhanced. Cell viability in the combination therapy of HT and ALA was 68.7 ± 5.3%, while control was 91.2 ± 4.7% (Figure 6B). This effect did not induce in normal cells (Figure 6C).

## 4. Discussion

In this study, we demonstrated that the combination of HT pretreatment and ALA can enhance the cytotoxicity of DOX in murine breast cancer cells. We previously demonstrated that the cytotoxic effect of PDT could be enhanced via regulation of the expression of transporters using rat gastric cancer cells. The expression of transporters was regulated via mitROS production. The expression of the ABC transporter was downregulated by the increase in mitROS production [20,27,28,29]. HT can increase the production of mitROS [20]; thus, we hypothesized that the cytotoxicity of DOX can enhance by HT. Moreover, we reported that the solute carrier (SLC) transporter was upregulated by the increase in mitROS production [30]. SLC15A1 is involved in the cellular uptake of ALA and is upregulated by mitROS. It is known that some anticancer drugs are taken into cells through SLC [16]. The DOX uptake transporter is an SLC protein known as SLC22A16. Therefore, we hypothesized and investigated whether HT would enhance the effects of anticancer drugs via the regulation of the expression of ABCG2 and SLC22A16.

We selected ALA as a competitive inhibitor. Porphyrin is a compound derived from ALA and is excreted by ABCG2. Thus, ALA acts as a competitive inhibitor for anticancer drugs, such as DOX, which are excreted by ABCG2. We previously reported that the increase in mitROS production upregulated the expression of SLC15A1 [30]. HT induces the production of mitROS and may accelerate uptake of ALA via SLC15A1. From these phenomena, we expected that HT is not only an inhibitor of porphyrin efflux but also an accelerator of ALA uptake.

In this study, we investigated whether the abovementioned effects could be induced in mouse breast cancer cells. Intracellular mitROS production was increased by HT treatment (Figure 1 and Figure 2). The HT treatment increased the accumulation of ALA-derived intracellular porphyrin as well as of DOX (Figure 3). We analyzed the transporter expression because intracellular mitROS production and porphyrin accumulation were increased by HT. HT treatment induced the downregulation of ABCG2 expression and the upregulation of SLC15A1 expression significantly; however, SLC22A16 expression was only slightly upregulated (Figure 4). These results indicated that mitROS regulates at least ABCG2 and SLC15A1 expression; however, it may not be involved in the regulation of SLC22A16 expression. Intracellular DOX accumulation was significantly increased by ALA or HT treatment. Moreover, fluorescence intensity of DOX in the combination treatment of ALA and HT was highest (Figure 5). The use of HT or ALA alone enhanced the cytotoxicity of DOX. The combination treatment with HT, ALA, and DOX accelerated the cytotoxicity (Figure 6A).

**Figure 7:**
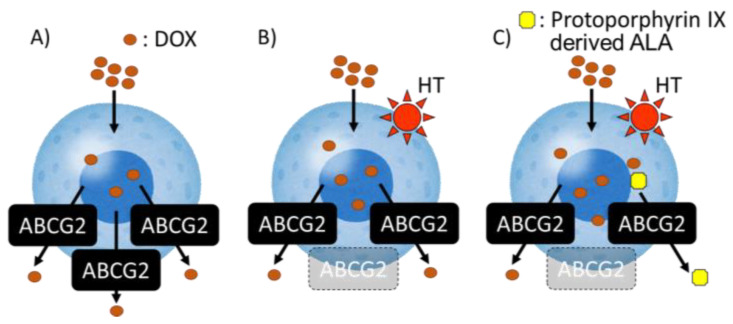
The mechanism of cell death in this study. (**A**) DOX is efflux from ABCG2. (**B**) HT can downregulate the ABCG2 expression. (**C**) Porphyrin is introduced by ALA and is excreted by ABCG2. ALA worked as a competitive inhibitor of DOX excretion transporter to enhance cell death.

One of the mechanisms in this treatment was the regulation of transporter expression. However, this treatment may affect an immune system or apoptosis signal. In this study, we utilized HT not as a direct treatment, but as a support treatment to regulate transporter expression. HT pretreatment accelerated the intracellular DOX accumulation and enhanced the cytotoxicity. We also investigated whether the combination therapy of both HT and ALA accelerates the effect of DOX in human cell line. MDA-MB-231 is a triple negative breast cancer cell line. The cytotoxicity of 1 μM DOX treatment in MDA-MB-231 enhanced by combination therapy with HT and ALA significantly (Figure 6B). Thus, we concluded that this combination therapy was more effective. This effect did not occur in normal cells (Figure 6C). From these results, we found new potential for a combination therapy of HT and chemotherapy in cancer specificity. It is reported that PDT using ALA enhances the cytotoxicity of DOX. Casas et al. showed that the enhancement of a PDT effect could be induced by attenuation of the cellular defense mechanisms involved in the increase in ROS production by DOX pretreatment [31]. Diez et al. reported that combination therapy of DOX and ALA–PDT significantly enhanced the cytotoxicity through synergistic effects [32]. In this study, we used ALA as a competitive inhibitor for DOX and showed that the cytotoxicity of DOX can be enhanced without PDT only by the addition of ALA (Figure 7).

## 5. Conclusions

The regulation of transporter expression by HT enhances the cytotoxicity of DOX. Moreover, in combination with ALA treatment, used as a competitive inhibitor, the cytotoxic effect of DOX is enhanced. The increase in intracellular porphyrin accumulation after ALA uptake is a cancer-specific phenomenon [28]; thus, competitive inhibition of an anticancer drug by ALA can result in fewer side effects for normal tissues. A combination of HT and chemotherapy was evaluated in a clinical trial [10,33,34]. The administration of ALA has no limitation in a clinical cure because it has no side effects. DOX is a standard chemotherapy drug in breast cancer. HT is also a treatment for breast cancer. Thus, in combination with HT, DOX and ALA can be very useful in future breast cancer treatments.

## Data Availability

Data is contained within the article.
